# Intestinal dysbiosis as an intraoperative predictor of septic complications: evidence from human surgical cohorts and preclinical models of peritoneal sepsis

**DOI:** 10.1038/s41598-023-49034-z

**Published:** 2023-12-21

**Authors:** Daniel Spari, Simone N. Zwicky, Bahtiyar Yilmaz, Lilian Salm, Daniel Candinas, Guido Beldi

**Affiliations:** Department of Visceral Surgery and Medicine, Inselspital, Bern University Hospital, University of Bern, Freiburgstrasse 18, 3010 Bern, Switzerland

**Keywords:** Experimental models of disease, Translational research, Microbiology

## Abstract

Major surgery exposes the intestinal microbiota to inflammatory and antibiotic stressors, which alter the microbiota composition of the intestinal lumen and fecal contents. However, it is not sufficiently understood, if such dysbiosis develops already during surgery and if alterations in microbiota may be the cause of surgical complications. End-of-surgery composition of the microbiota in the rectum was assessed in 41 patients undergoing either rectal or duodenopancreatic resection and was compared to baseline before surgery using 16S-rRNA sequencing. A subset of patients developed severe dysbiosis at the end of surgery, which was characterized by an overgrowth of the Proteobacteria phylum that includes the facultative pathogen *E. coli*. To test if dysbiosis impacts on surgical outcomes, dysbiosis was modeled in mice by a single oral administration of vancomycin prior to cecal ligation and puncture. Dysbiosis was associated with impaired post-surgical survival, dysregulation of the host’s immune response, elevated bacterial virulence and reduced bacterial metabolism of carbon sources. In conclusion, dysbiosis can be detected already at the end of surgery in a fraction of patients undergoing major surgery. Modelling surgery-associated dysbiosis in mice using single-shot administration of vancomycin induced dysbiosis and resulted in elevated mortality.

## Introduction

During major surgery, patients are exposed to multiple stressors such as perioperative antibiotics and surgery-induced inflammation that potentially induce changes of the intestinal microbiota composition, referred here as intestinal dysbiosis^1^. In the postoperative course after colorectal^2–4^, pancreatic^5^ or bariatric^6^ procedures but also transplantation^7^, intestinal dysbiosis was observed. Furthermore, the occurrence of such dysbiosis in patients with infectious and septic complications after surgery indicates an association with outcome.

Intestinal dysbiosis is characterized by a reduced abundance of obligate anaerobic bacteria, mainly members of the phyla Bacteroidota and Firmicutes, and an increase of facultative anaerobic bacteria of the Proteobacteria phylum, including the facultative pathogens *E. coli* and *Klebsiella spp.*^8^, both frequently found in organs and infected wounds after surgery^9^. It is well accepted that intestinal dysbiosis is associated with local and systemic inflammation^10,11^ as well as bacterial translocation^12^. However, it remains unclear if dysbiosis is only a consequence or also a driver of complications in surgical patients.

Since these previous studies analysed merely fecal samples, it remains unclear at what time point dysbiosis developed, given the interval of several days between surgery and the first bowel movement. Many infectious complications have its onset during rather than after surgery. Thus, to distinguish cause from consequence, it is critical to determine if dysbiosis is developing already during surgery^13^. In addition, feces represents only a snapshot of the intestinal microbiota and may rather poorly represent the mucosa-associated changes of the resident microbiota^14^. For instance, intraoperative specimens from the colon mucosa showed much stronger overgrowth of *Enterobacteriaceae* and *Enterococcus spp.* compared to postoperative fecal samples^2^. Thus, the analysis of postoperative fecal samples is unlikely to reflect the local alterations due to inflammation or anti-infective measures that occur in a well-specified but short time window during surgery.

Recently, rapid changes in the intestinal microbiota composition e.g. in response to overnight fasting or a meal were observed in samples obtained from ileostomies^15^. Thus, it may be hypothesized that alterations in microbiota composition and function occur during surgery and that such alterations may be relevant for the development of surgical infections.

In the current study, we assessed if changes of the intestinal microbiota can already be identified at the end of a surgical procedure. We therefore determined individual changes in the rectal microbiota of patients immediately at the end of major surgery and compared the findings to preoperative baseline. Two cohorts of patients undergoing major surgery were included: (1) patients undergoing rectal resection were included as a cohort with a high risk of septic complications and (2) patients undergoing duodenopancreatic resection were included as a cohort without manipulation of the large intestine. Based on the finding that the Proteobacteria phylum is increased at the end of surgery, single gavage of vancomycin was used to model such dysbiosis in mice^16^ and outcome was assessed in a model of cecal ligation and puncture (CLP) to represent septic surgical complications.

## Results

### Patients undergoing rectal and duodenopancreatic surgery exhibit intraoperative intestinal dysbiosis

**Figure 1:**
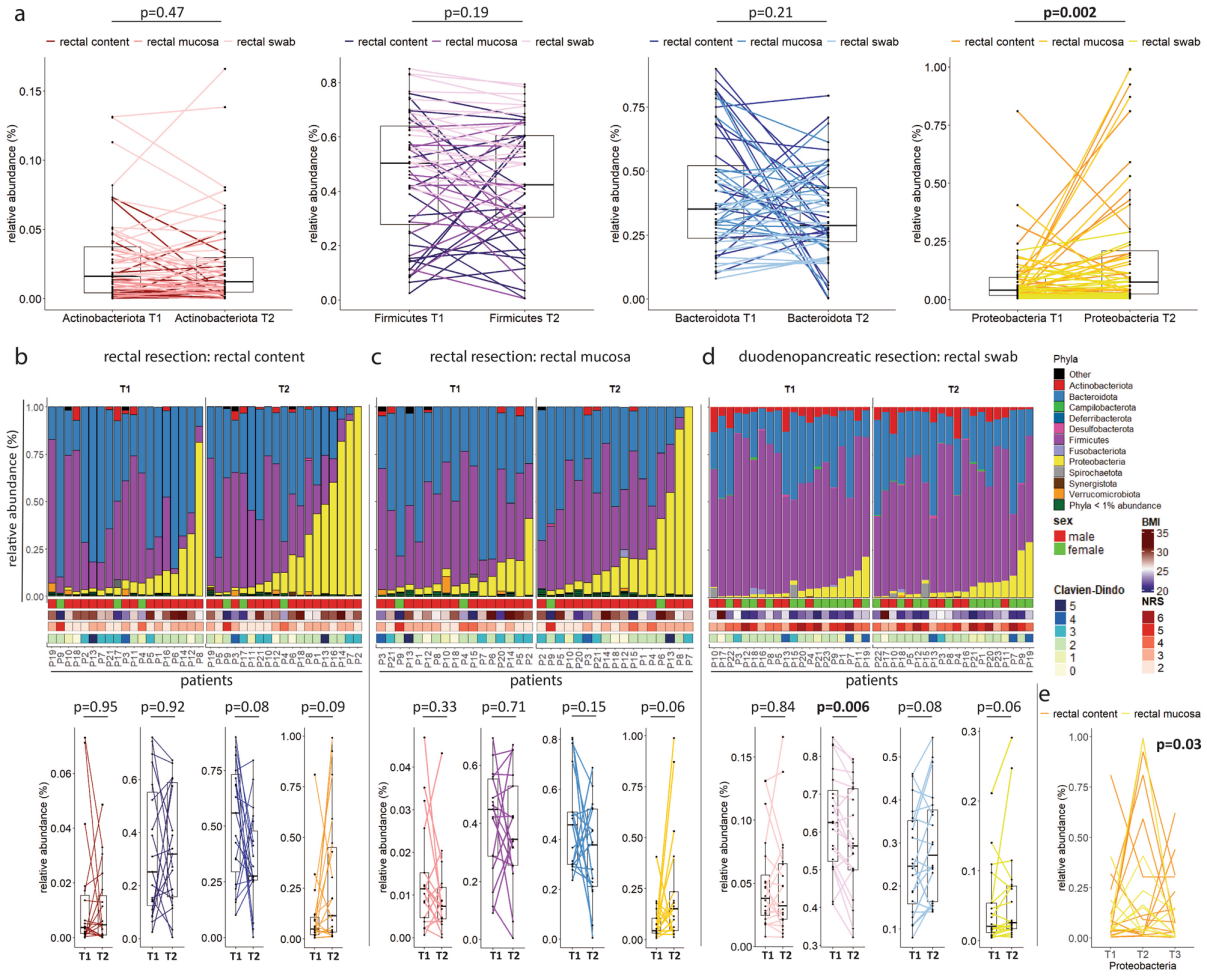
Dysbiosis in patients undergoing rectal and duodenopancreatic resection is characterized by a rapid expansion of Proteobacteria. Changes of the microbiota composition of the rectum was analysed using 16S-rRNA sequencing. In patients undergoing rectal resection, rectal luminal content and rectal mucosa (both: n = 15, luminal content only: n = 4, mucosa only: n = 2, total: n = 21) were obtained before surgery by rectoscopy (-50 to -6 days, (T1)) and immediately at the end of surgery from the surgical specimens (T2). In patients undergoing duodenopancreatic resection (total: n = 20), rectal swabs were obtained at the beginning of surgery (T1) and immediately at the end of surgery (T2). (**a**) Line plots of relative abundances of dominant bacterial phyla at T1 versus T2. Wilcoxon signed-rank test, n = 19 for rectal luminal content, n = 17 for rectal mucosa, n = 20 for rectal swabs. (**b**) Relative abundance barplots of rectal luminal content microbiota from patients undergoing rectal resection at T1 versus T2. Patient characteristics and relative abundances of dominant bacterial phyla at T1 versus T2 as line plots below. Wilcoxon signed-rank test, n = 19. (**c**) Relative abundance barplots of rectal mucosa microbiota from patients undergoing rectal resection at T1 versus T2. Patient characteristics and relative abundances of dominant bacterial phyla at T1 versus T2. Wilcoxon signed-rank test, n = 17. (**d**) Relative abundance barplots of rectal swab microbiota from patients undergoing duodenopancreatic resection at T1 versus T2. Patient characteristics and relative abundances of dominant bacterial phyla at T1 versus T2 as line plots below. Wilcoxon signed-rank test, n = 20. (**e**) Transient changes of relative abundances of the Proteobacteria phylum between T1, T2 and the first postoperative time point (T3, 15–61 days after surgery). Friedman test, n = 23; all patients with a T3 sample in addition to T1 and T2 sample were inclued. Boxes represent median and interquartile ranges (IQR); whiskers extend to a maximum of 1.5 IQR beyond the box.

To determine changes of the intestinal microbiota during surgery, rectal luminal content and rectal mucosa were obtained preoperatively by rectoscopy and at the end of surgery from surgical specimens obtained in patients undergoing rectal resection. In a second cohort, rectal swabs were collected in patients that underwent duodenopancreatic resection. Preoperative (T1) samples were compared with samples collected immediately at the end of surgery (T2) for each patient individually. A total of 41 different patients were analysed (Table 1). Among the dominant phyla, a significant increase in the Proteobacteria phylum was observed at T2 when compared to T1 (Fig. 1a, yellow line plot). The increase was stronger in patients undergoing rectal resection when compared to patients undergoing duodenopancreatic resection (Fig. 1b-d, yellow bars, and differentially shaded yellow line plots below). Community diversity was significantly different in rectal contents between T1 and T2 (Supplementary_figures, SFigure 1a-f). Deeper taxonomic analysis revealed that within the Proteobacteria phylum mainly members of the *Enterobacterales* order and the *Enterobacteriaceae* family expanded (Supplementary_figures, SFigure1g-i). *Enterobacteriaceae* include facultative pathogens such as *E.coli* or *Klebsiella spp.,* which are major causative bacteria for septic surgical complications. While there was a non-significant association of Proteobacteria with increased BMI and elevated nutritional risk score (NRS), there was neither an association with sex, age or incidence of complications (Fig. 1b-d), nor with bowel lavage or radiochemotherapeutic approaches (Table 1). Analysis of the intestinal microbiota in patients undergoing rectal resection at the late time point T3 that corresponds to first postoperative follow-up (Fig. 1e) indicates that surgery-associated intestinal dysbiosis is a transient phenomenon. In summary, intestinal dysbiosis emerged immediately but transiently at the end of surgery and was characterized by a strong increase of the Proteobacteria phylum, predominantly in patients who underwent rectal resection.

**Table 1:** Baseline characteristics and outcome parameters of the surgical cohorts.

Variable	Rectal resection (n = 21)	Duodenopancreatic resection (n = 20)
Age, mean years (SD)	66.3 (9.4)	66.8 (10.7)
BMI, mean kg/m^2^ (SD)	26.2 (3.8)	24.9 (6.0)
Female, n (%)	4 (19.0)	12 (60.0)
Comorbidities		
ASA score, mean (SD)	3.0 (0.4)	2.9 (0.4)
Smoking, n (%)	5 (23.8)	6 (30.0)
Diabetes, n (%)	2 (9.5)	4 (20.0)
Arterial hypertension, n (%)	13 (61.9)	12 (60)
Renal insufficiency, n (%)	4 (19.0)	1 (5.0)
NRS, mean (SD)	2.8 (0.8)	4.3 (1.1)
Tumor, n (%)	21 (100)	16 (80.0)
Neoadjuvant therapy		
Chemotherapy, n (%)	1 (4.7)	4 (20.0)
Chemoradiotherapy, n (%)	9 (42.8)	0
Short-course radiotherapy, n (%)	3 (14.3)	0
Surgical approach		
Open, n (%)	1 (4.7)	20 (100)
Laparoscopic, n (%)	11 (52.3)	0
Robotic, n (%)	9 (42.8)	0
Perioperative factors		
Bowel cleansing, n (%)	14 (66.6)	0
Duration of surgery, mean minutes (SD)	366.1 (98.6)	322.3 (75.3)
Blood loss, mean ml (SD)	246.6 (194.8)	492.5 (340.7)
Outcome parameters		
LOS, mean days (SD)	12.7 (8.9)	16.1 (3.9)
Clavien-Dindo Complication ≥ 3a, n (%)	8 (38.0)	5 (25.0)
CCI, mean (SD)	45.80 (26.7)	43.5 (15.4)
SSI, n (%)	8 (38.0)	8 (40.0)
30-day mortality, n (%)	1 (4.7)	0

*BMI* body mass index, *ASA* American Society of Anesthesiologists, *NRS* nutritional risk score, *LOS* length of stay, *CCI* comprehensive complication index, *SSI* surgical site infection.

### The *Enterobacteriaceae* family is dominant in superficial surgical site infections of patients undergoing duodenopancreatic resection

**Figure 2:**
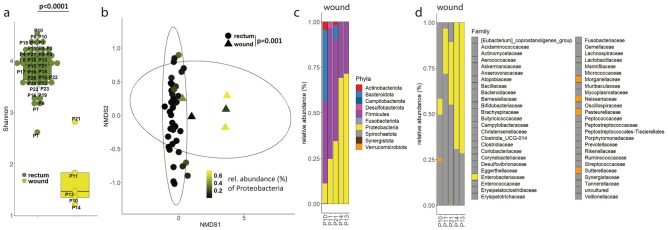
Samples of infected wounds from patients undergoing duodenopancreatic resection have low bacterial diversity and high abundance of Proteobacteria phylum. The microbiota composition of wound samples in patients with superficial surgical site infections after duodenopancreatic resection was analysed using 16S-rRNA sequencing. (**a**) Sample diversity (Shannon) in infected wounds (n = 5) compared to rectal swabs (T1 and T2, n = 40). Wilcoxon rank-sum test. Boxes represent median and interquartile ranges (IQR); whiskers extend to a maximum of 1.5 IQR beyond the box. (**b**) Community diversity (Bray–Curtis based non-metric multidimensional scaling (NMDS)) of infected wounds compared to rectal swabs (T1 and T2). PERMANOVA. Ellipses represent 95% confidence level of data points. (**c**) Relative abundance barpolot of infected wound swabs. (**d**) Deeper taxonomic classification (Family level) for Proteobacteria phylum.

Among the twenty patients who underwent duodenopancreatic surgery, five developed a superficial surgical site infection. Within these wound samples, 16S-rRNA sequencing indicated as expected that bacterial diversity was lower compared to rectal swabs (Fig. 2a). The bacterial community in the wound was enriched in Proteobacteria (Fig. 2b,c). Deeper taxonomic analysis indicates that the Proteobacteria phylum consisted mainly of the *Enterobacteriaceae* family (Fig. 2d).

### Modelling dysbiosis using oral vancomycin application

**Figure 3:**
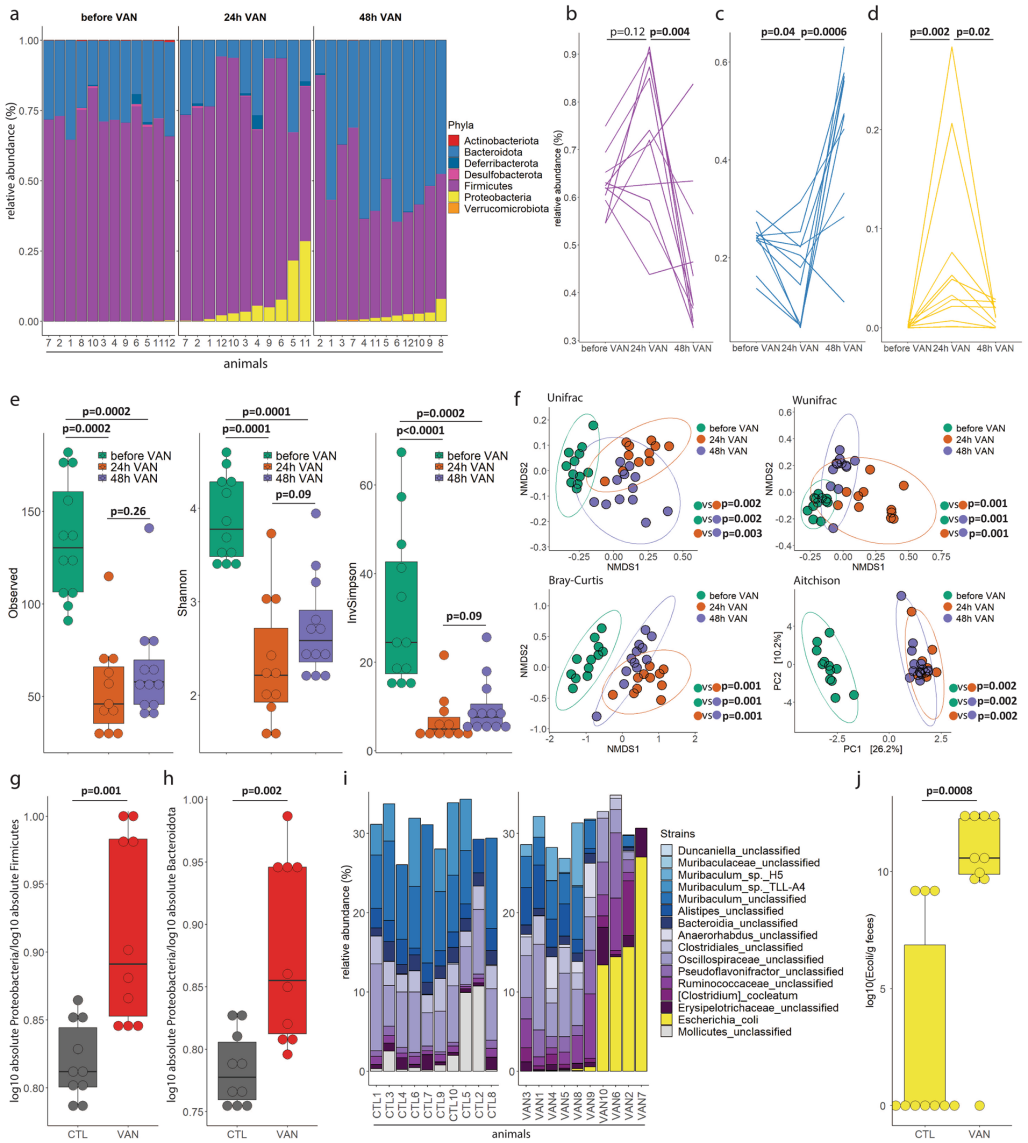
Model of transient dysbiosis using a single oral application of vancomycin. In 12 mice, 16S-rRNA sequencing was performed before, 24 h and 48 h after oral administration of 0.15 mg vancomycin. (**a**) Relative abundance barplots of bacterial phyla at the indicated time points. (**b**–**d**) Line plots of relative abundances of (**b**) Firmicutes, (**c**) Bacteroidota and (**d**) Proteobacteria phyla. Friedman test followed by pairwise Wilcoxon signed-rank test with Benjamini-Hochberg (BH) correction, n = 12. (**e**) Sample diversities (Observed, Shannon, InvSimpson) at the indicated time points. Kruskal–Wallis test followed by pairwise Wilcoxon rank-sum test, n = 12. (**f**) Community diversities (Unifrac, Wunifrac, Bray–Curtis, Aitchison) at the indicated time points. PERMANOVA with BH correction, n = 12. Ellipses represent 95% confidence level of data points. (**g**–**h**) Deeper sequencing using long-read 16S-ITS-23S sequencing. Expansion of the biomass of Proteobacteria over (**g**) Firmicutes and (**h**) Bacteroidota in the vancomycin (VAN) group compared to the control (CTL) group. Wilcoxon rank-sum test, n = 10 per group. (**i**) Proteobacteria phylum is mainly composed of *Escherichia coli (E. coli)* as assessed by Pacbio long-reading sequencing. (**j**) Absolute biomass of *E. coli* as assessed by flow cytometry with counting beads. Wilcoxon rank-sum test, n = 10 per group. Boxes represent median and interquartile ranges (IQR); whiskers extend to a maximum of 1.5 IQR beyond the box.

Given these rapid microbial changes during surgery, we established a mouse model of transient intestinal dysbiosis. A single oral gavage of vancomycin (VAN) was given to C57Bl/6 wild type mice to induce dysbiosis^16–18^. After this single dose, the relative abundance of Proteobacteria increased and peaked at 24 h (Fig. 3a,d). This was accompanied by a decrease of Bacteroidota (Fig. 3a,c). The relative abundance of Firmicutes did not change significantly within the first 24 h (Fig. 3a,b). Sample diversity (Fig. 3e) strongly decreased and community diversity (Fig. 3f) indicated strong differences at 24 h and 48 h compared to baseline before vancomycin administration. To identify changes of specific bacterial species, cecal contents were analysed using long-read 16S-ITS-23S sequencing (Fig. 3g-j). In contrast to control (CTL) mice, biomass of Proteobacteria increased significantly in comparison to Firmicutes (Fig. 3g) and Bacteroidota (Fig. 3h) in the VAN group. Sequencing indicated that such dysbiosis is mainly the result of expansion of *E.coli* (Fig. 3i,j). Taken together, single oral administration of vancomycin induces intestinal dysbiosis in C57Bl/6 wild type mice characterized by an expansion of *E.coli*.

### Dysbiotic cecal content increases septic complications

**Figure 4:**
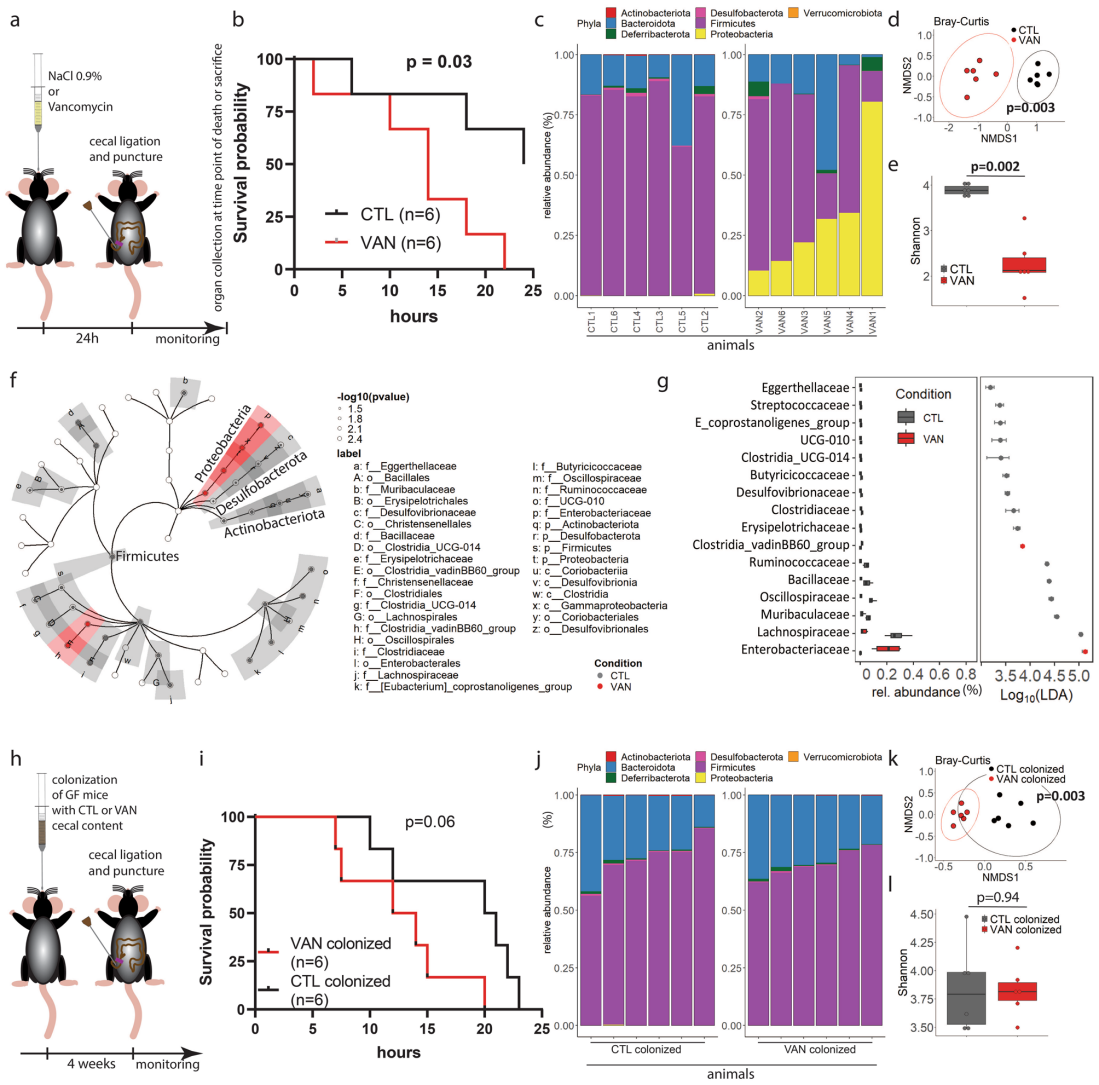
Dysbiosis in response to vancomycin is characterized by a bloom of the Proteobacteria phylum and leads to impaired survival after peritoneal sepsis. (**a**) Experimental approach for oral application of vancomycin 24 h prior to cecal ligation and puncture (CLP) and corresponding results (**b**–**g**). (**b**) Kaplan–Meier curves (log-rank test, n = 6 per group), (**c**) cecal microbiota composition, (**d**) cecal community diversity (PERMANOVA, n = 6 per group) and (**e**) sample diversity (Wilcoxon rank-sum test, n = 6 per group). (**f**) Cladogram of most differentially expressed bacterial taxa between CTL and VAN mice (n = 6 per group) and (**g**) relative abundance boxplot on the family level (LDA effect size (LEfSe), n = 6 per group). (**h**) Experimental approach for colonization of germ-free mice with either control (CTL-colonized) or vancomycin (VAN-colonized) cecal content four weeks prior to CLP and corresponding results (**i**–**l**). (**i**) Kaplan–Meier curves (log-rank test, n = 6 per group), (**j**) cecal microbiota composition, (**k**) cecal community diversity (PERMANOVA, n = 6 per group) and (**l**) sample diversity (Wilcoxon rank-sum test, n = 6 per group). Ellipses represent 95% confidence level of data points. Boxes represent median and interquartile ranges (IQR); whiskers extend to a maximum of 1.5 IQR beyond the box.

Severe infectious complications after major surgery can result from spillage of bowel contents after intestinal perforation, anastomotic leakage or intraabdominal abscesses and lead to high mortality^19^. Outcome after such infections is mainly dictated by the severity of peritoneal sepsis^20^. Thus, we aimed to assess, if dysbiosis determines postoperative outcomes using a model of CLP that generates polymicrobial abdominal sepsis. To assess the direct impact of a dysbiotic intestinal microbiota on survival, CLP was performed 24 h after pre-treatment with vancomycin (Fig. 4a), at the peak of Proteobacteria overgrowth (see Fig. 3a,d), and was compared to saline. Survival was significantly impaired in the VAN group (Fig. 4b) along with decreased sample and community diversity and high abundance of Proteobacteria (Fig. 4c-e). Analysis of differentially abundant bacterial taxa between these two groups revealed that Proteobacteria as well as the Clostridia vadin BB60 group were significantly more abundant in the VAN group (Fig. 4f). On the family level, the presence of *Enterobacteriacea* was the strongest predictor for impaired sepsis survival with a linear discriminant analysis (LDA) effect size of > 5 (Fig. 4g). To test if the vancomycin-induced changes are exclusively dependent on the intestinal microbiota, long-term colonisation with dysbiotic intestinal microbiota was assessed. For this purpose, germ-free mice were colonised with normal cecal contents from CTL mice or dysbiotic cecal contents from VAN mice followed by CLP (Fig. 4h). Survival of animals after engraftment of single-shot vancomycin treated donors was reduced but not significantly different (Fig. 4i). Importantly, overgrowth of the Proteobacteria phylum was not detectable four weeks after engraftment (Fig. 4j-l). These experiments indicate that preoperative vancomycin impairs survival and that engraftment of dysbiosis is not stable, underpinning the transient character of intestinal dysbiosis of this model.

### Dysbiotic cecal content induces profound changes of the immune milieu and is associated with metabolic changes and upregulation of virulence pathways in the microbiota

**Figure 5:**
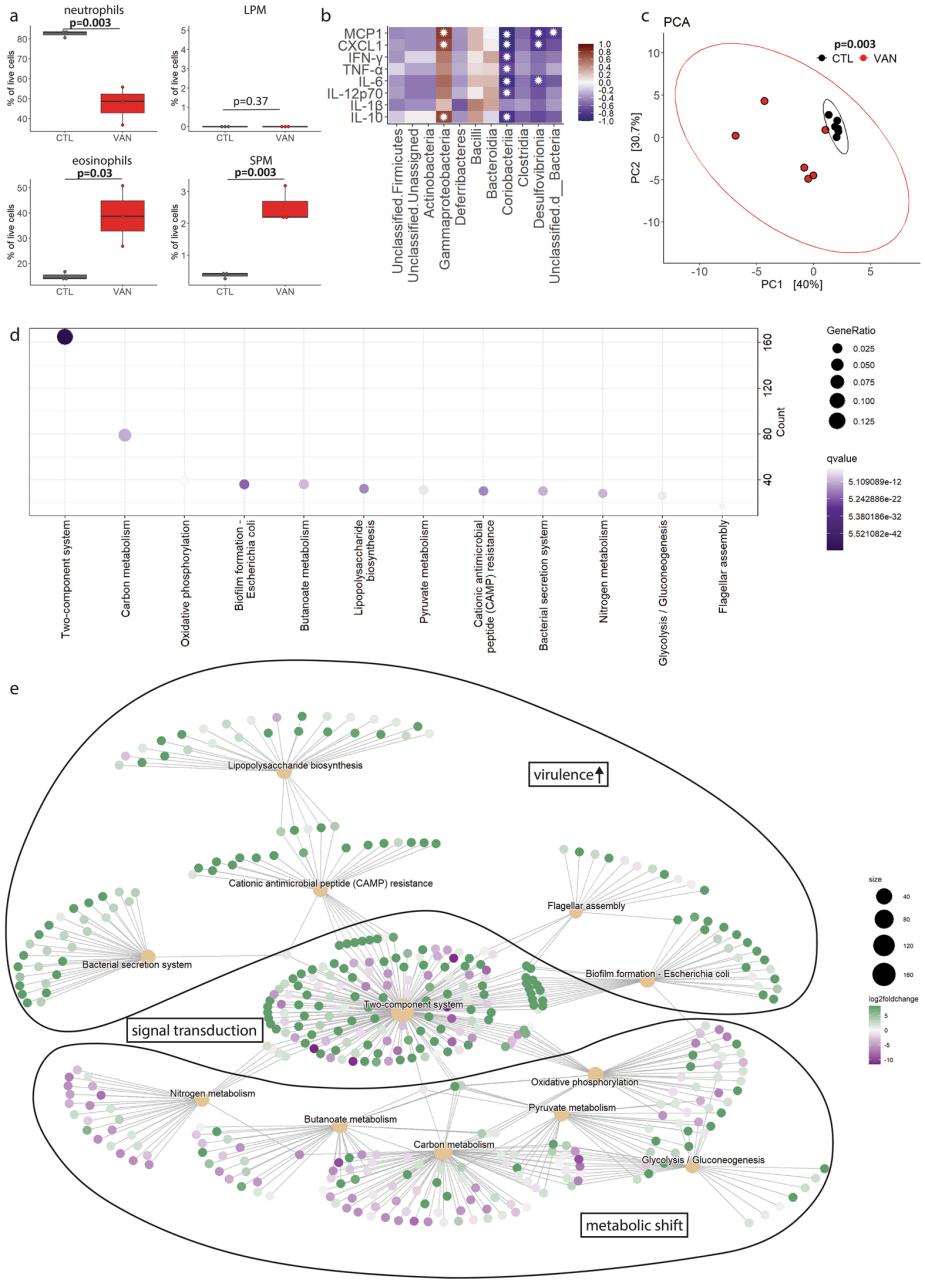
Dysbiosis induces altered immune responses and is associated with critical changes in bacterial virulence and metabolism. (**a**) Neutrophils, eosinophils, large peritoneal macrophages (LPM) and small peritoneal macrophages (SPM) in the peritoneal cavity were assessed 10 h after cecal ligation and puncture (CLP) in control (CTL) and vancomycin-treated (VAN) mice by flow cytometry. T-test, n = 3 per group. Boxes represent median and interquartile ranges (IQR); whiskers extend to a maximum of 1.5 IQR beyond the box. (**b**) Cross-correlation of cecal content microbiota and cytokines measured in the peritoneal cavity (see Fig. 4a-g). Spearman correlation with Benjamini-Hochberg (BH) correction, n = 12. Cross-correlations with a padj < 0.1 are marked with a white star. (**c**) Functional microbiota profile was inferred from cecal content microbiota of the CTL and VAN group (see Fig. 4a-g) using the PICRUSt2 pipeline. Principal component analysis indicates significant differences in the inferred KEGG orthologue abundances. PERMANOVA, n = 6 per group. Ellipses represent 95% confidence level of data points. (**d**, **e**) Enrichment analysis of inferred KEGG orthologue abundances (DESeq2, n = 6 per group).

To assess, how a dysbiotic intestinal microbiota influences postoperative outcome, cellular and soluble immune responses in the host and functional changes in the microbiota were analysed. In VAN mice that received single-shot vancomycin (see experimental approach Fig. 4a), peritoneal neutrophils were significantly reduced 10 h after CLP, whereas eosinophils were increased compared to CTL mice (Fig. 5a). Such cellular changes have been shown to be associated with impaired outcome after sepsis^21,22^. Furthermore, a cross-correlation of intestinal microbiota composition and cytokines measured in the peritoneal fluid at the harvesting time point after CLP (see Fig. 4a-g) was performed. The *Gammaproteobacteria* class, which is highly abundant, was significantly positively associated with cytokines that are often dysregulated in sepsis, like IL-10, MCP-1, CXCL-1 (Fig. 5b)^23^. To predict functional changes based on altered cecal content microbiota composition, the PICRUSt2 pipeline was applied^24^. This resulted in a table of inferred KEGG orthologue abundances per sample^25–27^. Principal component analysis of these KEGG orthologues indicates a significant difference in gene profiles between the two groups (Fig. 5c). Enrichment analysis revealed significant differences in gene expression and associated pathways between CTL and VAN mice (Fig. 5d,e, Supplementary_tables STable 1–13). These pathways indicate that dysbiosis was associated with activation of bacterial signal transduction inducing elevated virulence and metabolic adaptation towards anaerobic glycolysis at the expense of the metabolism of short chain fatty acids, nitrogen and carbon sources. Taken together, vancomycin-induced dysbiosis led to a dysregulated cellular host immune response and was associated with cytokine dysregulation as well as increased bacterial virulence and impaired bacterial metabolism of carbon sources.

## Discussion

The present study reveals that immediately at the end of surgery, typical features of intestinal dysbiosis^28^ with overgrowth of the Proteobacteria phylum, mainly the *Enterobacteriaceae* family, are present in a subset of patients. Identifying changes of the intestinal microbiota composition immediately at such an early time point indicates that future studies need to focus on end-of-surgery dysbiosis.

Modelling this clinical observation in mice indicates that dysbiosis impairs peritonitis-associated survival. This preclinical model therefore shows that dysbiosis is a cause of complications rather than only its consequence. The fact that the same families of facultative pathogens that expanded in the rectum during surgery were identified in infected wounds suggests also a potential association of dysbiosis with remote infections.

In this study, we were mainly focusing on the perioperative microbiota changes themselves and not (yet) on the potential factors that lead to surgery-induced dysbiosis. Such potential factors for dysbiosis include (1) availability of antibacterial factors such as oral or systemic antibiotics and patient-derived intestinal antimicrobial peptides, (2) availability of bacterial nutrients such as short-chain fatty acids, glycans, nitrates, bile acids^29^ or luminal as well as epithelial oxygen^28^, potentially contributing to the bloom of oxygen-consuming Proteobacteria at the expense of facultative and obligate anaerobic Firmicutes and Bacteroidota or (3) inflammation and altered integrity of the mucosal layer^30^. The results of the mouse model indicate that there are notable changes in the bacterial metabolism. This is supported by the observation that epithelial lactate concentrations during cardiac surgery were strongly elevated intra-rectally up to 16 h after surgery^31^. Furthermore, in the fasting patient, glycans derived from the epithelium can promote the expansion of bacteria having the machinery to digest mucins^32^. As surgery is typically performed in patients after an overnight fasting, endogenous molecules attached to cell surfaces, shed epithelial cells and secreted mucus are available to be metabolized by commensal microbes^33^. Together, such surgery-dependent changes of the epithelial and/or luminal metabolism are likely to immediately impact on the intestinal bacterial composition.

Limitations of the clinical studies are their exploratory design with a limited number of patients. The studies were also not powered to identify an association with a clinical outcome such as systemic infection or inflammation. However, the clinical data are highly relevant for the precise design of future clinical trials. Proteobacteria are well recognized as an effector of nosocomial infections such as superficial surgical site infections^34^. Whether such a high abundance in wounds as observed in the present study is related to the expansion in the rectum remains to be analysed. Nevertheless, the finding raises very important and interesting questions, which need to be addressed in future studies using full bacterial genome sequencing to identify characteristics that might be important for systemic translocation of bacteria and to explore surgery-dependent impairments of the microbial compartmentalization.

The limitations of the experimental studies are intrinsic to the use of oral vancomycin. The studies indicate that dysbiosis is a potential effector of outcome but cannot exclude off-target effects of vancomycin such as neutropenia, eosinophilia, altered autophagy or cytokine release^35,36^. However, oral vancomycin is hardly absorbed at physiological dosages as used in these studies limiting the systemic effects^37^. Another limitation of the mouse model are the differences in baseline human and mouse microbiota composition.

Our observations raise many important questions to be addressed in future studies. For example, it will be interesting to determine if and how the basal microbiota structure dictates the incidence of dysbiosis. Also, how such rapid dysbiosis drives complications needs to be addressed. It is unknown if bacterial translocation already occurs, e.g. to local lymph nodes and to systemic organs such as the lung or to superficial wounds. It may also be questioned if expanding bacteria within the intestine secrete metabolites that exacerbate or diminish surgery-related local or systemic inflammation and modulate host’s immune response.

In conclusion, the study reveals that intestinal dysbiosis is detectable at the end of major surgery. Modelling similar patterns of microbiota disruption in a rodent model of surgical peritoneal sepsis demonstrates significantly impaired survival for dysbiotic animals indicating a relationship between intestinal dysbiosis and outcome in patients undergoing major surgery. These hypothesis-generating studies are the basis for future targeted investigations.

## Methods

### Patient data

Two single-center cohorts of patients were analysed. One cohort included patients undergoing elective rectal resection. The human experimental protocol was approved by the Kantonale Ethikkommission Bern, Switzerland (ethical approval 2017-00573, NCT03554148). From these patients, either rectal luminal content, rectal mucosa-associated tissue or both, were collected preoperatively (− 50 to − 6 days) (T1), immediately at the end of surgery (T2) and at the first postoperative follow-up (15 to 61 days) (T3). Patients who had adjuvant cancer therapy within 4 weeks before T3 were excluded. This resulted in a total of 21 patients. A second cohort of total 23 subsequent patients undergoing elective duodenopancreatic resection was analysed. The human experimental protocol was approved by the Kantonale Ethikkommission Bern, Switzerland (ethical approval 2019-00576, NCT04096885). Rectal swabs were collected directly before surgery (T1) and at end of surgery (T2). Among the 23 patients, three patients were excluded. Two patients underwent surgery other than duodenopancreatic resection and one patient had insufficient reads. Furthermore, all superficial wound infections from patients who underwent duodenopancreatic resection were sampled.

All samples from both cohorts were directly snap frozen and analysed by 16S-rRNA sequencing as described below. All patients of the two cohorts were fasted overnight and none of them received antibiotics within three weeks before operation. Bowel preparation was done using one liter of Moviprep in a subset of the rectal resection cohort. All rectal resection patients received a combination of i.v. Co-Amoxicillin, Metronidazol and Garamycin as perioperative antibiotic prophylaxis, whereas duodenopancreatic resection patients received i.v. Cefuroxim. No oral antibiotic decontamination was used. Written informed consent was obtained from all patients and both studies have been performed in accordance to the Declaration of Helsinki as well as the CONSORT guidelines. Patients’ demographics, therapeutic and surgical features, as well as outcome parameters were extracted from an electronical database.

### Mouse handling

Specific-pathogen-free (SPF) C57BL/6J RccHsd mice were purchased at the age of 8 weeks from Envigo (Netherlands) and were housed in ventilated cages in the central animal facility, University of Bern, Switzerland. All experiments were performed in the morning, mice were supplied with a 12-h light/dark cycle at 22 °C and fed ad libitum with chow and water. Since mixing of mice is paramount to establish a baseline microbiota over several cages, only female mice were used and the ordered batch of mice was mixed upon arrival until three days before the experiment between cages. Germ-free wild-type C57BL/6J animals were bred and housed in flexible film isolators at the Clean Mouse Facility of the University of Bern, Switzerland. Germ-free status was confirmed by aerobic and anaerobic culture as well as DNA stain using SYTOX green of fecal contents to detect unculturable contamination. All animal procedures were carried out in accordance to the Swiss guidelines for the care and use of laboratory animals as well as in accordance to the ARRIVE guidelines and were approved by the Animal Care Committee of the Canton of Bern (Switzerland) under the following number: BE41/2022.

### Vancomycin application

To induce dysbiosis, mice were orally gavaged with 0.15 mg non-resorbable vancomycin dissolved in 300 µl saline, 24 h before intervention. Controls were gavaged with 300 µl saline only.

### Sample collection

To assess microbiota dynamics, fecal pellets were sampled directly from the mouse into a sterile 2 ml tube before, 24 h and 48 h after vancomycin application. Fecal samples were snap frozen, stored at -80 °C until DNA extraction and analysed by 16S-rRNA amplicon sequencing. For strain-level characterization of microbiota, fresh cecal content was collected anaerobically (5% H_2_, 10% CO_2_, 85% N_2_) 24 h after vancomycin or saline application. Samples were snap frozen, stored at -80 °C until DNA extraction and analysed by StrainID PacBio amplicon sequencing. To characterize gut microbiota of VAN colonized, CTL colonized, VAN and CTL mice that underwent CLP, cecal contents were collected anaerobically (5% H_2_, 10% CO_2_, 85% N_2_) when animals had to be euthanized according to the monitoring and latest after 24 h.

### Absolute quantification of bacteria

Cecal samples were weighed and diluted in a known amount of sterile saline. Bacterial cells were stained using the Cell Viability Kit with BD Liquid Counting Beads according to the manufacturer’s protocol. Stained bacteria and beads were acquired on a CytoFLEX S and analysis was done with FlowJo software. Bacterial biomass was determined and calculated back to bacteria/g feces according to the manufacturer’s protocol.

### Animal model of peritoneal sepsis (CLP)

Twenty-four hours after vancomycin or saline application, CLP was performed as described elsewhere^38^. In brief, mice were anesthetized s.c. injecting a mixture of fentanyl, dormicum and medetor and were then shaved and disinfected with Betadine. Mid-line laparotomy was performed (approx. 1 cm) and the cecum was exposed. The proximal 1/3 of the cecum were ligated with Vicryl 4–0 and perforated with a 23 G needle. The cecum was returned to the peritoneal cavity and the laparotomy was sutured continuously in two layers with prolene 6–0. At the end, the antidote (naloxone, revertor, temgesic) was s.c. injected. A semi-quantitative score sheet was used to predict animal postoperative well-being. Mice were evaluated every four hours according to the following criteria: appearance, level of consciousness, activity, response to stimulus, eye shape, respiratory rate and respiratory quality and analgesia was applied if necessary. If the score reached specific criteria, the animal was sacrificed using pentobarbital followed by organ collection.

### Colonization of germ-free mice

Fresh cecal content was collected anaerobically (5% H_2_, 10% CO_2_, 85% N_2_) 24 h after gavage from VAN and CTL mice. Approximately 100 µg cecal content was homogenized in 200 µl sterile O_2_-reduced saline and administered by oral gavage to germ-free mice. Colonization status was assessed by 16S-rRNA sequencing of cecal content collected at the harvesting time point after CLP.

### Cytokine assay

The peritoneal cavity was flushed with 1 ml of saline, the mouse was gently massaged, and the aspirated fluid was collected. Cytokines in the fluid were measured using a customized U-Plex mesoscale cytokine assay according to the manufacturer’s protocol. The cytokines included were INF-γ, IL-1β, IL-2, IL-4, IL-6, IL-10, IL-12p70, KC, MCP-1 and TNF-α.

### Flow cytometry

The peritoneal cavity was flushed two times with 5 ml MACS buffer (DPBS supplemented with 3% FBS, 2% HEPES and 2 mM EDTA) and aspirated fluid was spun at 700 g for 5 min. to pellet the peritoneal cells. Cells were washed one time with MACS buffer. Next, live/dead dye (see METHODS TABLE) together with Fc-block was diluted in DPBS and cells were incubated for 20 min. at 4 °C in the dark. Cells were washed with MACS buffer and surface staining was done with the listed antibody cocktail (see METHODS TABLE) for 20 min. at 4 °C in the dark. Again, cells were washed with MACS buffer and resuspended in MACS buffer for acquisition on the LSR-Fortessa. Analysis was done with FlowJo software.

### DNA extraction

DNA from patients undergoing rectal resection was extracted using the QIAamp Fast DNA Stool Mini Kit according to the manufacturer’s protocol and with the following modifications: 100 mg fecal content was homogenized in 500 μl Buffer ASL by bead-beating (Retsch MM300 Tissue Lyser at 30 Hz for 3 min.), followed by a 95 °C heat-based lysis step. After repeating the bead-beating and heating step, samples were incubated with 200 μl Lysis Buffer (20 mg/ml lysozyme; 20 mM Tris–HCl, pH 8.0; 2 mM EDTA; 1.2% Triton) for 30 min. This step allowed us to increase the DNA yield for difficult to lyse intestinal microbiota components^39^.

DNA from rectal swabs from patients undergoing duodenopancreatic resection was extracted using the QIAamp PowerFecal Pro DNA Kit according to the manufacturer’s protocol. DNA was stored at -80 °C until downstream processing.

### 16S-rRNA amplicon sequencing

The V5/V6 region of 16S-rRNA genes was amplified using KAPA HiFi HotStart ReadyMix starting from 10 to 50 ng template DNA using the following primers^40^:

- forward 5′-CCATCTCATCCCTGCGTGTCTCCGACTCAG-barcode-ATTAGATACCCYGGTAGTCC-3′.
- reverse 5′-CCTCTCTATGGGCAGTCGGTGATACGAGCTGACGACARCCATG-3′.

amplifying an expected product of ~ 350 bp^41^. The following PCR conditions were used: initial 5 min. at 95 °C denaturation, followed by 30 cycles of 20 s denaturation at 98 °C, 20 s annealing cycle at 60 °C, and 20 s extension cycle at 72 °C, with a final extension for 7 min. at 72 °C. PCR products run on 2% gel for 90 min. cut out and purified using Qiaquick Gel Extraction Kit. The amplicon concentration was determined using Qubit 3.0 Fluorometer and 26 pM of each sample were pooled into a library tube. Sequencing was carried out using the Ion PGM Sequencing 400 Kit and Ion 316TM Chip V2 within the IonPGM System.

Raw reads were processed on the UBELIX Linux cluster of the University of Bern using the QIIME2 pipeline^15,42^. First, raw reads were imported using *qiime tools import* and demultiplexed using *qiime cutadapt demux-single*. Then, reads were trimmed using *qiime cutadapt trim-single*, quality-controlled using *qiime demux summarize* and denoised using *qiime dada2 denoise-single*. A phylogenetic tree was produced using *qiime phylogeny align-to-tree-mafft-fasttree* and the reads were aligned using a trained silva classifier using *qiime feature-classifier classify-sklearn*. QIIME2 artifacts were downstream processed using a custom R script keeping only samples with more than 1000 reads (exclusion of one duodenopancreatic resection patient sample).

### Pacbio StrainID amplicon sequencing

DNA was amplified using dual-barcoded primers targeting 16S-ITS-23S, (Shoreline Biome, Shoreline Wave, StrainID Set). First, a single-step PCR using barcode and target-specific primers was done to generate amplicons ready for PacBioSMRTbell template preparation. Subsequently, samples were sequenced on the PacBio Sequel Systems. The whole protocol was done according to the Shoreline Wave for PacBio Technical Manual. The pooled amplicons were quantified (Qubit, see above) and their length was verified using an Advanced Analytical Fragment Analyzer System and a Fragment Analyzer NGS Fragment Kit.

Barcoded SMRTbell libraries were constructed using an Express TPK 2.0 in conjunction with the Barcoded Overhang Adapter Kit 8A & 8B. This was performed according to the Shoreline Protocol for High-Density Multiplexing on PacBio Sequel Systems. Again, the quantity and size of each SMRTbell library was checked using Qubit and a Fragment Analyzer. Two barcoded SMRTbell libraries were sequenced on a PacBio SMRT cell 8 M. For polymerase binding, the PacBio Sequel II 2.1 binding kit was used, together with a Sequel II 2.0 sequencing kit as well as Sequel II DNA Internal Control. Thereafter, CCS sequencing mode and a 10 h moving time was used. The reads were sorted into the original sample pools by demultiplexing the SMRTbell barcodes using PacBio SMRTLink v9.0 software after each run. After SMRTbell demultiplexing and primer removal, the pooled amplicon files were again demultiplexed into individual samples. Taxonomy was assigned to all reads using the SBAnalyzer software (v3.1) and Athena database (v2.2) using the StrainID_Kit.txt pipeline. Downstream analysis was done using a custom R script.

### PICRUSt2 pipeline

PICRUSt2 is a tool to predict functional abundances based on marker gene sequences. The following command was used to generate the data^24^:

- *picrust2_pipeline.py -s dna-sequences.fasta -i feature-table.biom -o picrust2_out_pipeline -t epa-ng -m mp -verbose*

### Statistical analysis

All statistical analyses were performed in R except the survival analyses, which were done in Graphpad Prism (log-rank test). For differences between two groups a t-test was applied when data was normally distributed (parametric), otherwise a Wilcoxon rank-sum test was used (non-parametric). For paired data, a paired t-test or Wilcoxon signed-rank test was performed respectively. For differences between more than 2 groups, a one-way ANOVA followed by a tukey post-hoc test (parametric), or a Kruskal–Wallis test followed by a pairwise Wilcoxon rank-sum test with Benjamini-Hochberg (BH) correction (non-parametric) was used. To compare 3 groups that are paired (repeated measurements) and not normally distributed, a Friedman test followed by a pairwise Wilcoxon signed-rank test with BH correction was applied. No one-tailed test was used. Beta diversity was calculated with the function pairwise.adonis() (PERMANOVA) from the “pairwiseAdonis” R package with BH correction. This approach was also used for principal component analysis with *sim.method* = *“euclidean”* after normalization *(microbiome::transform( …, method* = *“standardize”, …)*. For differential expression analysis of bacteria, *diff_analysis()* (followed by *ggdiffclade()* or *ggdiffbox()*) of the “MicrobiotaProcess” R package was applied which also provides an effect size measure (Linear Discriminant Analysis (LDA))^43^. Enrichment analysis of picrust-derived KEGG Orthologies (KO) was done in a stepwise process. First, differential abundance of KO was calculated with *DESeq()* from the “DESeq2” R package^44^. Then significantly differential abundant KO were enriched and plotted using *enrichKEGG()*, *dotplot()* and *cnetplot()* from “clusterProfiler” R package^45^. P < 0.05 (p =  = padj, when correction for multiple testing was necessary) was considered significant in all statistical analyses unless stated otherwise in the figure legend. Significant differences are in bold.

## Methods table

**Table:** 

REAGENT/SOFTWARE/RESOURCE	SOURCE	IDENTIFIER
Antibodies		
Purified anti-Ms CD16/32	Biolegend	Cat# 101302; clone 93; Lot# B298973; RRID: AB_312801
Rat anti-Ms F4/80 (BUV395)	BD Biosciences	Cat# 565614; clone T45-2342; Lot# 1104580; RRID: AB_2739304
Rat anti-Ms CD3 (eFluor450)	ThermoFisher Scientific	Cat# 48-0032-82; clone 17A2; Lot# 264580; RRID: AB_1272193
Rat anti-Ms Ly-6C (PerCP-Cyanine5.5)	ThermoFisher Scientific	Cat# 45-5932-82; clone HK1.4; Lot# 2309273; RRID: AB_2723343
Rat anti-Ms CD19 (Super Bright 600)	ThermoFisher Scientific	Cat# 63-0193-82; clone eBio1D3; Lot# 2366423; RRID: AB_2637308
Rat anti-Ms CCR2 (BV650)	Biolegend	Cat# 150613; clone SA203G11; Lot# B294599; RRID: AB_2721553
Rat anti-Ms I-A/I-E (BV711)	Biolegend	Cat# 107643; clone M5/114.15.2; Lot# B299330; RRID: AB_2565976
Mouse anti-Ms CX3CR1 (BV785)	Biolegend	Cat# 149029; clone SA011F11; Lot# B304744; RRID: AB_2565938
Rat anti-Ms Ly-6G (FITC)	BD Biosciences	Cat# 551460; clone 1A8; Lot# 9068981; RRID: AB_394207
Rat anti-Ms Siglec F (PE)	ThermoFisher Scientific	Cat# 12-1702-80**;** clone 1RNM44N; Lot# 2252684; RRID: AB_2637129
Armenian hamster anti-Ms FcεR1α (PE/Dazzle 594)	Biolegend	Cat# 134331 clone MAR-1; Lot# B280348; RRID: AB_2687240
Mouse anti-Ms NK-1.1 (PE-Cy7)	BD Biosciences	Cat# 552878 clone PK136; Lot# 9189638; RRID: AB_394507
Rat anti-Ms/Hs CD11b (APC)	Biolegend	Cat# 101212 clone M1/70; Lot# B312600; RRID: AB_312794
Rat anti-Ms CD102 (AF700)	SouthernBiotech	Cat# 1925-27 clone 3C4; Lot# F1912-SK45B; RRID: AB_2795545
Armenian hamster anti-Ms (APC-eFluor780)	ThermoFisher Scientific	Cat# 47-0114-80 clone N418; Lot# 2133269; RRID: AB_1548652
Chemicals peptides and recombinant proteins		
Vancocin (vancomycinum) Trockensub 500 mg i.v	Teva Pharma AG	N/A
Fentanyl (fentanylum) Sintetica 0.1 mg/2 ml	Sintetica SA	N/A
Dormicum (midazolamum) 15 mg/3 ml	CPS Cito Pharma Services GmbH	N/A
Medetor (medetomidin) ad us. vet	Virbac	N/A
Brain Heart Infusion Broth	Oxoid	Cat# CM0225
SYTOX Green Nucleic Acid Stain	ThermoFisher Scientific	Cat# S7020
Cell viability kit with BD liquid counting beads	BD Biosciences	Cat# 349480
Fixable Viability Dye eFluor506	ThermoFisher Scientific	Cat# 65-0866-18
Glycerol	Sigma-Aldrich	Cat# G5516-500 ml
DPBS (1x)	Gibco	Cat# 14190-094
HEPES buffer solution	Sigma-Aldrich	Cat# H0887-100 ml
EDTA disodium salt dihydrate	Sigma-Aldrich	Cat# E5134-500G
Tris-HCl	Sigma-Aldrich	Cat# T3253
Lysozyme	Sigma-Aldrich	Cat# 62970-5G-F
Triton X-100	Sigma-Aldrich	Cat# 93443
Foetal bovine serum	Gibco	Cat# 10500-064
KAPA HiFi HotStart ReadyMix	Roche	Cat# 7958935001
Critical commercial assays		
QIAamp Fast DNA Stool Mini Kit	Qiagen	Cat# 51604
QIAamp PowerFecal Pro DNA Kit	Qiagen	Cat# 51804
QIAquick Gel Extraction Kit	Qiagen	Cat# 28706
Qubit dsDNA HS Assay Kit	Thermo Scientific	Cat# Q32854
Ion PGM™ HiQ View Sequencing 400 Kit	ThermoFisher Scientific	Cat# A30044
U-PLEX assay	Meso Scale Discovery	Cat# K15069L-1
Small Fragment and NGS Kits	Agilent	Cat# DNF-473-1000
Shoreline Wave™ StrainID™ for PacBio	Shoreline Biome	Cat# WAVESID-A
SMRTbell Express		
Template Prep Kit 2.0	PacBio	Cat# 100-938-900
Barcoded overhang		
adapter kit 8A	PacBio	Cat# 101-628-400
Barcoded overhang		
adapter kit 8B	PacBio	Cat# 101-628-500
Sequel II 2.1 binding kit	PacBio	Cat# 101-843-000
Sequel II 2.0 sequencing kit	PacBio	Cat# 101-820-200
Experimental models: organisms/strains		
SPF C57BL/6 J RccHsd mice	Envigo	N/A
Germ-free C57BL/6 J mice	University of Bern Clean mouse facility	N/A
Oligonucleotides		
5′-CCTCTCTATGGGCAGTCGGTGATACGAGCTGACGACARCCATG-3′	Yilmaz B. et al. (2018)^40^	N/A
5′-CCATCTCATCCCTGCGTGTCTCCGACTCAG-BARCODE-ATTAGATACCCYGGTAGTCC-3	Yilmaz B. et al. (2018)^40^	N/A
Software and algorithms		
QIIME2 v2021.11	Bolyen E. et al. (2019)^42^	https://qiime2.org
*qiime tools import*	Bolyen E. et al. (2019)^42^	https://qiime2.org
*qiime cutadapt demux-single*	Martin M. (2011)^46^	https://qiime2.org
*qiime cutadapt trim-single*	Martin M. (2011)^46^	https://qiime2.org
		https://qiime2.org
*qiime dada2 denoise-single*	Callahan et al. (2016)^47^	https://qiime2.org
*qiime phylogeny align-to-tree-mafft-fasttree*	Katoh K. et al. (2002)^48^ Price M.N. et al. (2010)^49^	https://qiime2.org
*qiime feature-classifier classify-sklearn*	Robeson M. S. et al. (2020)^50^ Bokulich N.A. et al. (2018)^51^	https://qiime2.org
SBanalyzer™ v3.1	Shoreline Biome	https://shorelinebiome.com/resources/
Athena™ database v2.2	Shoreline Biome	https://shorelinebiome.com/sbanalyzer-software-onepager-download/
SMRTLink v9.0	Shoreline Biome	https://shorelinebiome.com/resources/
R v4.2.2	The R Project for Statistical Computing	https://cran.r-project.org
RStudio v2022.07.2.576	RStudio Desktop	https://www.rstudio.com
R package clusterProfiler v4.6.2	Wu T. et al. (2021)^52^	https://guangchuangyu.github.io/software/clusterProfiler
R package coin v1.4-2	Hothorn T. et al. (2006)^53^	https://cran.r-project.org/web/packages/coin/index.html
R package ComplexHeatmap v2.14.0	Gu Z. et al. (2016, 2022)^54,55^	https://jokergoo.github.io/ComplexHeatmap
R package DESeq2 v1.38.3	Love M.I. et al. (2014)^44^	https://github.com/mikelove/DESeq2
R package dplyr v1.0.10	Wickham H. et al. (2022)^56^	https://CRAN.R-project.org/package=dplyr
R package ggplot2 v3.4.3	Wickham H. (2016)^57^	https://ggplot2.tidyverse.org
R package ggpubr v0.6.0	Kassambara A. (2023)^58^	https://CRAN.R-project.org/package=ggpubr
R package ggrepel v0.9.3	Slowikowski K. (2023)^59^	https://CRAN.R-project.org/package=ggrepel
R package janitor v2.2.0	Firke S. et al. (2023)^60^	https://CRAN.R-project.org/package=janitor
R package KEGGREST v1.40.0	Tenenbaum D. Maintainer B. (2022)^61^	https://bioconductor.org/packages/release/bioc/html/KEGGREST.html
R package microbiome v1.20.0	Lahti L. et al. (2012)^62^	https://microbiome.github.io
R package MicrobiotaProcess v1.10.3	Xu S. Yu G. (2022)^63^	https://github.com/YuLab-SMU/MicrobiotaProcess
R package pairwiseAdonis v0.4	Martinez A. P. (2017)^64^	https://github.com/pmartinezarbizu/pairwiseAdonis
R package phyloseq v1.42.0	McMurdie P.S. Holmes S. (2013)^65^	https://github.com/joey711/phyloseq
R package qiime2R v0.99.6	Bisanz J. E. (2018)^66^	https://github.com/jbisanz/qiime2R
R package RColorBrewer v1.1-3	Neuwirth E. (2022)^67^	https://CRAN.R-project.org/package=RColorBrewer
R package readxl v1.4.3	Wickham H. Bryan J. (2023)^68^	https://CRAN.R-project.org/package=readxl
R package reshape2 v1.4.4	Hadley W. (2007)^69^	http://www.jstatsoft.org/v21/i12
R package rstatix v0.7.2	Kassambara A. (2023)^70^	https://CRAN.R-project.org/package=rstatix
R package scales v1.2.1	Wickham H. Seidel D. (2022)^71^	https://CRAN.R-project.org/package=scales
R package tibble v3.1.8	Müller K. Wickham H. (2022)^72^	https://CRAN.R-project.org/package=tibble
R package tidyr v1.3.0	Wickham H. et al. (2023)^73^	https://CRAN.R-project.org/package=tidyr
R package vegan v2.6-4	Oksanen J. et al. (2022)^74^	https://github.com/vegandevs/vegan
PICRUSt2 v2.4.2	Douglas G.M. et al. (2020)^24^	https://github.com/picrust/picrust2/wiki
EPA-NG v0.3.8	Barbera P. et al. (2019)^75^	https://github.com/Pbdas/epa-ng
Gappa v0.7.0	Czech L. et al. (2020)^76^	https://github.com/lczech/gappa
SEPP v4.3.10	Mirarab S. et al. (2012)^77^	https://github.com/smirarab/sepp
Castor v1.7.3	Louca S. et al. (2018)^78^	https://cran.r-project.org/web/packages/castor/index.html
FlowJo v10.8.1	FlowJo™ software	https://www.flowjo.com
Graphpad Prism v9.4.1	Prism Graphpad™ software	https://www.graphpad.com/
Deposited data		
16S-rRNA amplicon sequencing data	This paper	BORIS portal
Pacbio StrainID amplicon sequencing data	This paper	BORIS portal
Other		
Anaerobic chamber	Don Whitley Scientific	N/A
Mixed compressed gas n.o.s	Carbagas AG	Cat# UN1956
Retsch MM300 TissueLyser	Qiagen	N/A
TissueLyser II	Qiagen	Cat# 85300
Animal feeding needle curved 23G 4 cm steel barrel-shaped	N/A	N/A
Peripheral IV cannula	BD Insyte	Cat# 381223
Single-use needle 23G (0.6 × 30 mm)	BD Microlance 3	Cat# 300700
Single-use needle 26G (0.45 × 10 mm)	BD Microlance 3	Cat# 300300
Vicryl suture 4-0	Ethicon	Cat# V1224H
Prolene suture 6-0	Ethicon	Cat# MPP8697H
CytoFLEX S	Beckman Coulter	N/A
BD LSR-Fortessa Cell Analyzer	BD Biosciences	N/A
Nanodrop 2000	Thermo Scientific	Cat# ND2000
Qubit 3.0 Fluorometer	ThermoFisher Scientific	N/A
Advanced Analytical Fragment Analyzer System	Agilent	N/A
PacBio Sequel IIe	PacBio	N/A
Ion 316™ Chip Kit V2	ThermoFisher Scientific	N/A
IonPGM™ System	ThermoFisher Scientific	N/A

### Supplementary Information

Supplementary Information 1.

Supplementary Information 2.

## Data Availability

Ion Torrent 16S-rRNA and Pacbio StrainID amplicon sequencing data has been deposited in the European Nucleotide Archive (ENA) at EMBL-EBI under accession number PRJEB67798 (https://www.ebi.ac.uk/ena/browser/view/PRJEB67798). In addition, all sequencing data as well as all fully anonymized source data (except patient data), scripts, metadata and code are publicly available at BORIS portal of the University of Bern under a CC BY licence: 10.48620/372. Furthermore, 16S-rRNA sequencing data for patients that underwent rectal resection in Fig. 1, which was reanalysed from the following study: “Plasticity of the adult human small intestinal stoma microbiota. Cell Host Microbe, doi:10.1016/j.chom.2022.10.002”, is available at https://figshare.com/s/077130437ca8ac314386. A list of the used samples is available at BORIS portal named rectal_resection_samples_used.xlsx.
