# Supplementary material for: Intestinal dysbiosis as an intraoperative predictor of septic complications: evidence from human surgical cohorts and preclinical models of peritoneal sepsis
Source: Sci Rep. 2023 Dec 21;13:22921. doi: 10.1038/s41598-023-49034-z (PMC10739899; doi:10.1038/s41598-023-49034-z)
Supplement: Supplementary file 1 — Supplementary Information 1. [file 41598_2023_49034_MOESM1_ESM.pdf]

Intestinal dysbiosis as an intraoperative predictor of septic complications: evidence from human surgical cohorts and preclinical models of peritoneal sepsis

Daniel Spari[1], Simone N. Zwicky[1], Bahtiyar Yilmaz[1], Lilian Salm[1], Daniel Candinas[1], Guido Beldi[1*]

[1]Department of Visceral Surgery and Medicine, Inselspital, Bern University Hospital, University of Bern, Switzerland
[*]Corresponding author: Guido Beldi; guido.beldi@insel.ch; Freiburgstrasse 18, 3010 Bern

Supplementary Figure

SFigure 1: Sample and community diversities as well as taxonomic classification on the family level from all patient groups separated according to the two time points T1 and T2.

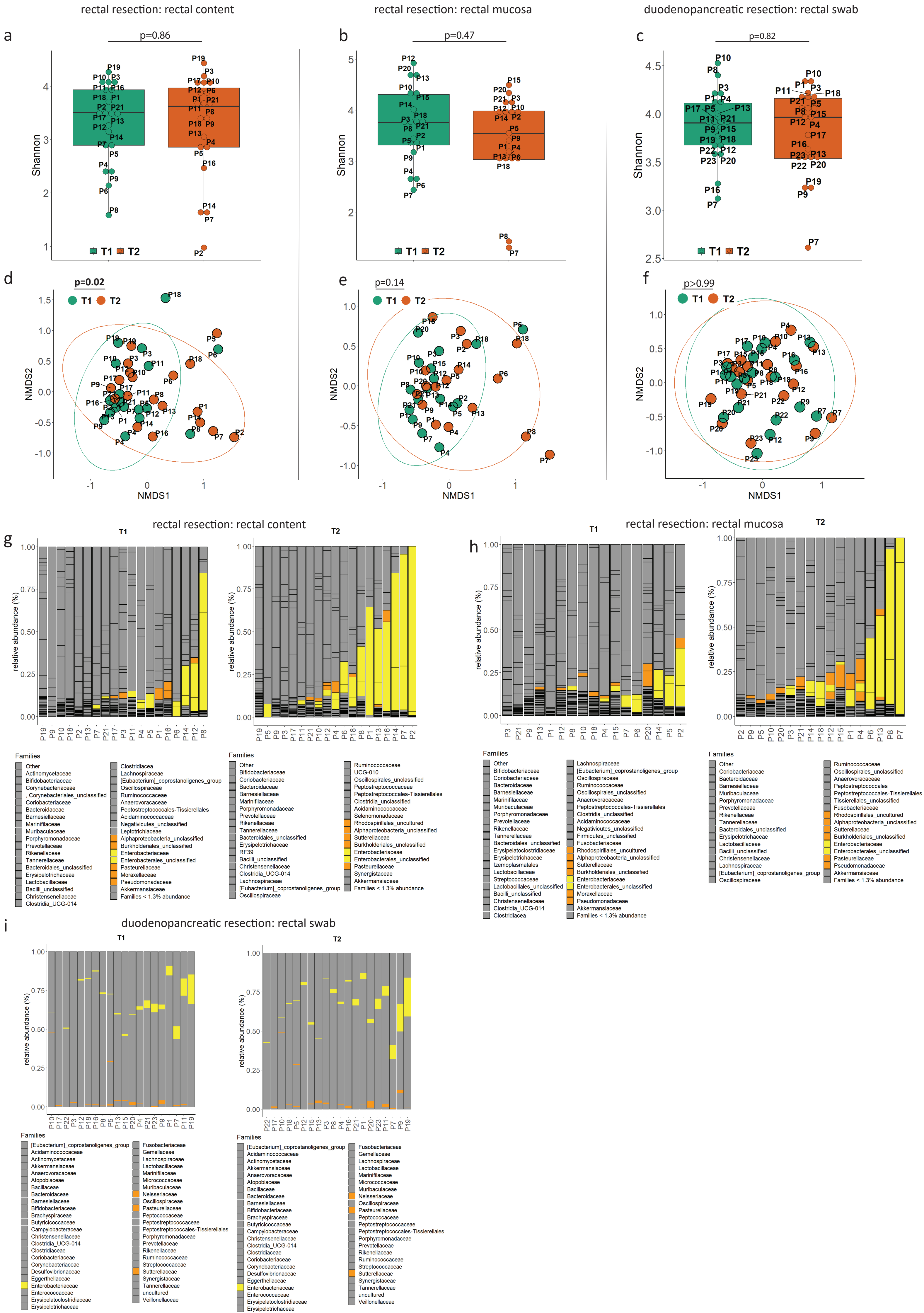

rectal resection: rectal content
rectal resection: rectal mucosa
duodenopancreatic resection: rectal swab
a
b
c
p=0.86
p=0.47
p=0.82
Shannon
T1
T2
d
e
f
p=0.02
p=0.14
p>0.99
NMDS1
NMDS2
g
rectal resection: rectal content
h
rectal resection: rectal mucosa
relative abundance (%)
Families
Other
Actinomycetaceae
Bifidobacteriaceae
Corynebacteriaceae
Corynebacteriales_unclassified
Coriobacteriaceae
Bacteroidaceae
Barnesiellaceae
Marinifilaceae
Muribaculaceae
Porphyromonadaceae
Prevotellaceae
Rikenellaceae
Tannerellaceae
Bacteroidales_unclassified
Erysipelotrichaceae
Lactobacillaceae
Bacilli_unclassified
Christensenellaceae
Clostridia_UCG-014
Clostridiaceae
Lachnospiraceae
[Eubacterium]_coprostanoligenes_group
Oscillospiraceae
Ruminococcaceae
Anaerovoracaceae
Peptostreptococcales-Tissierellales
Acidaminococcaceae
Negativicutes_unclassified
Leptotrichiaceae
Alphaproteobacteria_unclassified
Burkholderiales_unclassified
Enterobacteriaceae
Enterobacterales_unclassified
Pasteurellaceae
Moraxellaceae
Pseudomonadaceae
Akkermansiaceae
Families < 1.3% abundance
i
duodenopancreatic resection: rectal swab

SFigure 1: Changes in bacterial composition in patients undergoing rectal resection and duodenopancreatic resection.

a-c) Sample diversity (Shannon index) of rectal content, rectal mucosa and rectal swab samples at T1 vs. T2. Wilcoxon rank-sum test, n = 19 for a, n = 17 for b, n = 20 for c. Boxes represent median and interquartile ranges (IQR); whiskers extend to a maximum of 1.5 IQR beyond the box.

d-f) Bacterial community diversity (Bray-Curtis based non-metric multidimensional scaling (NMDS)) of rectal content, rectal mucosa and rectal swab samples at T1 vs. T2. PERMANOVA, n = 19 for d, n = 17 for e, n = 20 for f. Ellipses represent 95% confidence level of data points.

g-i) Deeper taxonomic classification shows that the Proteobacteria fraction mainly consists of the *Enterobacterales* order and the *Enterobacteriaceae* family, n = 19 for g, n = 17 for h, n = 20 for i.
